# Autotoxicity in *Panax notoginseng* of root exudatesand their allelochemicals

**DOI:** 10.3389/fpls.2022.1020626

**Published:** 2022-12-20

**Authors:** Wei Xiang, Jianhua Chen, Fengyuan Zhang, Rongshao Huang, Liangbo Li

**Affiliations:** ^1^ College of Pharmacy, Guangxi University of Chinese Medicine, Nanning, China; ^2^ College of Horticulture, Hunan Agricultural University, Changsha, China; ^3^ College of Agriculture, Guangxi University, Nanning, China

**Keywords:** *Panax notoginseng*, continuous cropping obstacle, root exudates, autotoxicity, allelochemicals

## Abstract

The growth of *Panax notoginseng* (Burk.) F. H. Chen is frequently hindered due to replanting failure. In the present study, the objective is to determine whether root exudates from *P. notoginseng* have autotoxicity and identification of allelochemicals from root exudates or rhizosphere soil. We investigated autotoxicity in *P. notoginseng* using seedling emergence bioassays and hydroponic culture. The allelochemicals in the soils and root exudates were identified with GC-MS, and the autotoxicity of the identified key allelochemicals was investigated by bioassay. The results showed that the root exudates, and extracts from consecutively cultivated soils also showed significant autotoxicity against seedling emergence and growth. In the non-renewed culture solution without activated charcoal (AC), the fresh and dry mass of *P. notoginseng* tubers of roots was reduced by about half compared to the addition with AC. A total of 44 different components from all samples were defined by GC-MS analyses. Furthermore, the results of multiple statistical analysis showed a t the difference among cultivated soil, uncultivated soil and root exudates. Bioassay of the identified allelochemicals revealed that benzoic acid, phthalic acid, palmitic acid, and stearic acid significantly affected the root growth of *P. notoginseng*. These substances at 100 μM more significantly decreased the number of lateral roots. Our results demonstrated that autotoxicity results in replant failure of *P. notoginseng*.

## 1 Introduction

*Panax notoginseng* (Burk.) F. H. Chen is a valuable medicinal material in China. In China, *P. notoginseng* has more than 400 years of artificial cultivation (Qiao et al., 2018). Due to its unique geographical environment, *P. notoginseng* is mainly distributed in Yunnan and Guangxi Province, China (Dong et al., 2003). Demand for ginsenosides has rapidly increased due to their significant effects on cancer and cardiovascular disease (Xu et al., 2018; Liu et al., 2020). However, *P. notoginseng* on the market mainly relies on artificial cultivation, with limited land resources, production is often hindered by replanting failure, leading to lower yields and other difficulties when reestablishing plants in arable land (Yang et al., 2015; Dong et al., 2018). Generally, rotation is the preferred method to avoid failure of crop replanting (Wang et al., 2022). However, the successful replanting of *P. notoginseng* replanting requires more than 30 years of rotation (Yang et al., 2018), which limit the production of *P. notoginseng*.

Many factors can contribute to this problem, including deterioration of soil physicochemical properties, nutrient imbalance, soil-borne diseases, and autotoxicity (Qiao et al., 2020). Currently, the research on the continuous cropping obstacles of *P. notoginseng* is mostly focused on the treating and preventing pests and diseases. Diseases were caused by many types of soil-borne pathogens established, including fungi, oomycetes, bacteria, nematodes, and viruses (Jin et al., 2019; Fang et al., 2022; Wang et al., 2022). However, even using of specific fungicides, satisfactory results have not been achieved (Liu et al., 2019; Ye et al., 2019). Therefore, continuous cropping obstacles have become a major problem limiting the development of the *P. notoginseng* industry.

Previously, plant autotoxicity was reported to be one of the main factors causing continuous cropping obstacles (Singh et al., 1999). Autotoxicity is a common phenomenon in natural and agroecosystems, where toxins were released by living or decaying plants into their surroundings, thus lead to inhibition of the growth and development of the same species (Inderjit and Duke, 2003). In crop root exudates or rhizosphere soil, allelochemicals accumulate over years of cultiva, causing nutrient imbalances and microbial dysfunction in the soil, which cause continuous cropping obstacles (Li et al., 2019). The autotoxicity of *Panax ginseng* and *Panax quinquefolius* has been reported as a possible factor for transplant failure. (Zhang et al., 2020; Ji et al., 2021). Some phenolic acids in American ginseng’s root exudate and rhizosphere soil have been evaluated as potential allelochemicals (He et al., 2009). Ferulic acid, total saponin, and root extract of *P. notoginseng* could inhibit the plant’s growth (Yang et al., 2015). The crop can be grown continuously on the same land for years when the inhibitory chemicals are removed. (Gao and Wen, 2016; Xiang et al., 2020). Therefore, it is very important to understand the compositions of these inhibitory chemicals.

Studying on the autotoxicity of *P. notoginseng* would provide effective guidance for crop sustainable production. The objective of this study was to (i) confirm autotoxicity of root exudates in *P. notoginseng*, and (ii) identify allelochemicals therein.

## 2 Materials and methods

### 2.1 Determination of biological activities of soil extracts

Soil samples were collected in Baise city, Guangxi County (23°34 ′ 11 ″ N, 105°55 ′ 56 ″ E). The rhizosphere soil samples were obtained from fields that had been cultivated with continuous cropping for 1 to 3 years. Soil samples were collected from an adjacent uncultivated land as a control. At each collection site, nine samples were collected randomly and mixed thoroughly before being divided into three. Take 150 g of the air-dried and sieved soil samples, and put them in a triangular flask. After adding 100 mL of distilled water, ultrasonically extract for 30 min. The obtained solution was filtered with filter paper, and the extracts were collected. The above steps were repeated two times for the remaining samples, and all the extracts were mixed. The solvent was removed by vacuum rotary evaporation at 55°C to obtain the extracts. The extracts were dissolved in distilled water, and the volume was made up to 100 mL, and the concentration of 1.5 g/mL (that is, the extract containing 1.5 g of soil sample in 1 mL aqueous solution) was prepared as a stock solution. Two layers of filter paper were placed in Petri dishes (9 cm in diameter), and 5 mL of distilled water or soil extracts were added, respectively, and three replicates were set up for each treatment. Select the same size and disease-free *P. notoginseng* seeds, these seeds were sterilized with 1% NaClO for 15 min and then washed three times with sterilized water. Twenty seeds were placed in each dish and then put in a constant temperature incubator, cultivated at 25°C without light, ventilated once a day, ensured that the filter paper was wet and determine the germination rate after 30 d (Yang et al., 2015).

### 2.2 Plant cultivation either with or without AC

The autotoxicity of *P. notoginseng* was researched using hydroponics with or without activated charcoal (AC). The *P. notoginseng* used in the study was collected in Guangxi County (23°34 ′ 11 ″ N, 105°55 ′ 56 ″ E). The plants were transplanted into the seedling pots (50 cm × 25 cm × 15 cm) in a greenhouse of Guangxi University. Twelve plants were grown in each pot, with three replicates per treatment. Each pot was filled with an 8 L nutrient solution, and the nutrient solution was updated every 15 d. The nutrient solution uses the general formula of Hoagland and Arnon (Zandvakili et al., 2019). Each pot has two air pumps with filters for continuous aeration (3.5 L/min), and each filter contains 100 g AC. The control group was treated equally. The AC is used for chemicals secreted from plants, and at the same time, it is replaced with fresh AC for 2 weeks until the end of the experiment. The used AC was either immediately stored at 4°C for later extraction. Relevant agronomic traits were measured at the end of the experiment. Also, three plants from each treatment were taken to test the enzymatic activities of root viability, the enzyme activities of catalase (CAT), peroxidase (POD), and malondialdehyde (MDA) in leaves (Md. Asaduzzaman, 2012).

### 2.3 GC–MS analysis of root exudates adsorbed in AC and soil

Extracted components in AC or soils samples with reference to previous reports (Md. Asaduzzaman, 2012). One microliter of the concentrated sample was injected into a GC-MS system (Agilent GC7890A/MSD5975C, USA). The GC conditions were as follows: carrier gas, helium; splitless mode; temperature programming, 60°C (1 min), 60-180°C (10°C/min), and 180-280°C (20°C/min). Separation was achieved on a HP-5MS 5% Phenyl Methyl Silox column (30 mm × 0:25 mm × 0:25 μm). Mass spectra were obtained at an electron impact (EI) of 70 eV (Ren et al., 2017).

### 2.4 Autotoxicity bioassays

In order to further determine the autotoxicity of the precipitated chemical substances. Allelochemical aqueous solutions of different concentrations were prepared with 50% Hoagland nutrient solution (Md. Asaduzzaman, 2012). The selected plants were transplanted to seedling trays, each hole containing one plant, and an equal amount of vermiculite was added to each hole to hold the plants tight and upright. The trays were placed in a greenhouse at 25°C with a light intensity of 2000 Lux and 16 h photoperiod. Every 30 d, 20 mL of test solution was added to each hole, and each treatment was replicated 20 times. The plants were grown for two months and then the plant length, root length, and the number of lateral roots were measured.

### 2.5 Statistical analysis

The growth and yield data obtained from bioassay and hydroponics of plants were compiled and analyzed for statistical differences among the treatments and means were separated by the analysis of variation with Duncan’s test, LSD test, and t-test using SPSS Statistics 24. The multivariate data (GC-MS) analyses were conducted using MetaboAnalyst 4.0 (www.metaboanalyst.ca). To confirm an overview of clustering separation between different exposure groups, a partial least squares-discriminant analysis (PLS-DA), which is a supervised pattern recognition method, was conducted to distinguish between each treatment group, and R 2 Y and Q 2 parameters were also calculated. The VIP (variable importance in the projection) in PLS-DA was calculated to select metabolites with VIP scores > 1.

## 3 Results

### 3.1 Effects of aqueous extract from consecutively cultivated soil on seedling germination and growth

**Figure 1:**
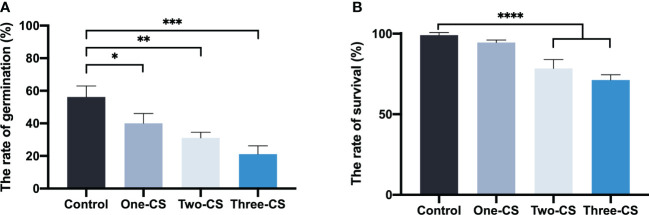
Effects of aqueous extract from consecutively cultivated soil on seedling germination rate **(A)**, and seedling survival rate **(B)**. Control represents the aqueous extract from the uncultivated soil, and One-CS, Two-CS, and Three-CS represent the aqueous extract from one, two, and three years of continuously cultivated soil. Values presented are means. Error bars represent the standard error of three replicates. Asterisks indicate statistically significant differences of treatment compared with control. *, p < 0.05; **, p < 0.01; ***, p < 0.005; ****, p < 0.001, LSD test.

Compared with the control (aqueous extract from the uncultivated soil), the aqueous extract from different consecutively cultivated soils inhibited seed germination and seeding emergence to different degrees (**Figure 1**). Moreover, the germination rate declined significantly with the increase of successive planting years. The germination rate only reached 21.1% when the seeds were treated with aqueous extract from soil that was continuously cultivated for three years. Three months after added different aqueous extracts from soils in the seeding tray, the seedling survival rate in the control was 99.1%. However, the seeding survival rate treated with the aqueous extracts from soils that were consecutively cultivated for two and three years was reduced significantly to about 20.79% and 28.05%, with control, respectively.

### 3.2 Growth of *P. notoginseng* in hydroponics

**Figure 2:**
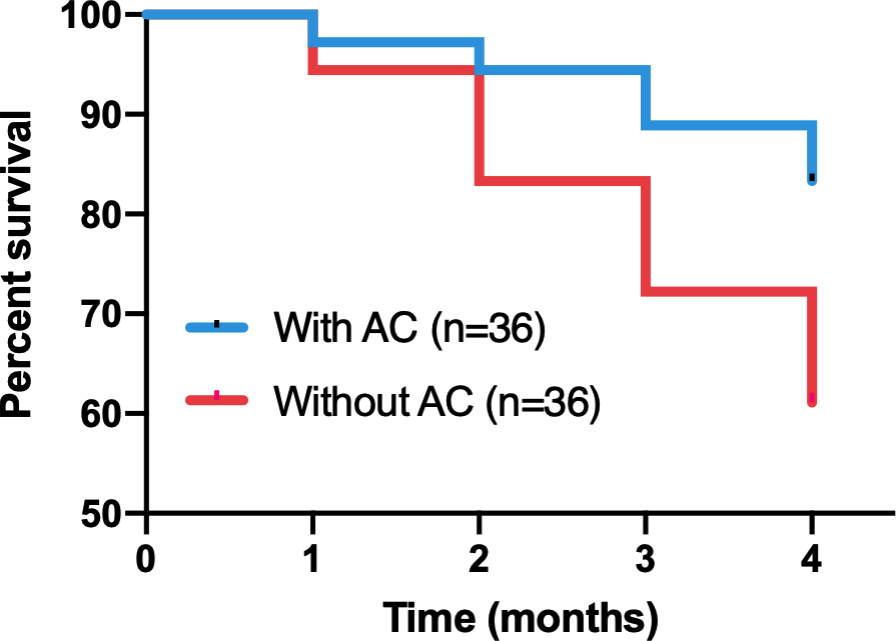
Survival curves of *P. notoginseng* either with or without AC addition in hydroponics. Through Kaplan-Merier with log-rank test (Prism).

**Figure 3:**
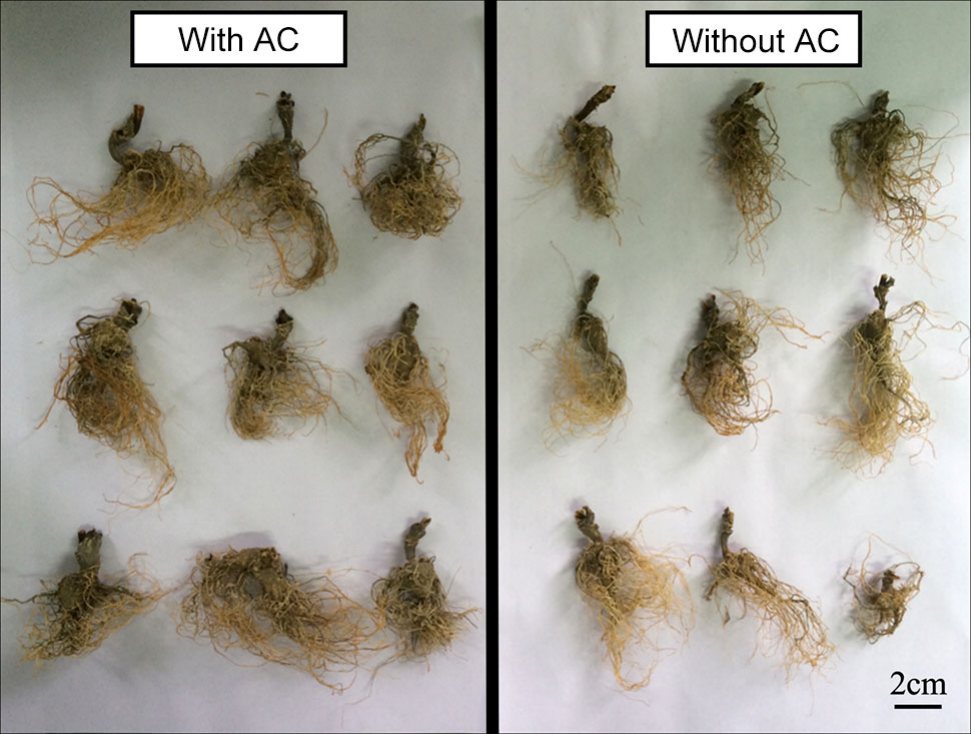
Dry root of *P. notoginseng* either with or without AC addition in hydroponics.

Culture solutions containing AC had significant effects on the growth of *P. notoginseng*. The survival rate declined significantly(Log Rank P=0.0352)in the plants grown without AC compared with those grown with AC (**Figure 2**). After the fourth month of observation, in control (without AC) plants, the plant length, and their fresh and dry mass of aboveground in *P. notoginseng* were reduced to about 1.57%, 19.35%, and 17.95% of the values compared with AC, respectively. There was a significant difference in fresh and dry mass of roots between plants cultivated with and without AC addition, and the values increased by about 72.6% and 88.5% compared with the control, respectively (**Table 1**, **Figure 3**).

**Table 1:** Growth of *P. notoginseng* either with or without AC addition in hydroponics.

Treatment	Plant length (cm)	FM^1^ of above ground(g)	FM of root(g)	DM^1^ of above ground(g)	DM of root(g)
Without AC	27.42 ± 1.38	3.25 ± 0.22	5.22 ± 0.41	0.64 ± 0.04	1.31 ± 0.12
With AC	27.86 ± 1.43	4.03 ± 0.37	9.01 ± 0.40 **	0.78 ± 0.08	2.47 ± 0.23 **

^1^ FM Fresh mass (FM), dry mass (DM). Note: Values presented are means ± SE. SE represent the standard error of three replicates. Asterisks indicate statistically significant differences of treatment compared with control (Water). ** p< 0.01, T-test (SPSS).

### 3.3 Effects of root exudates on the physiological indexes of *P. notoginseng*

The results for antioxidant enzymes (CAT, POD, and MDA) activity and root activity of these plants were shown in **Table 2**, the activities of CAT, POD, and MDA were 33.02%, 22.42%, and 11.73% lower than those of the control group, and the root activity was 6.59% higher than that of the control group. However, the difference in these values between the two groups was not considered statistically significant (p<0,05).

**Table 2:** Physiological indexes of *P. notoginseng* either with or without AC addition in hydroponics.

Treatment	CAT	POD	MDA	Root vitality
	(U·g^-1^Fw·min^-1^)	(U·g^-1^Fw·min^-1^)	(mmol·g^-1^Fw)	(ug·g Fw·h)
Without AC	12.57 ± 3.30	3.88 ± 0.15	13.90 ± 0.94	197.33 ± 6.26
With AC	8.42 ± 2.30	3.01 ± 0.70	12.27 ± 1.04	210.33 ± 5.30

Values presented are means ± SE. SE represent the standard error of three replicates.

### 3.4 Identification of allelochemicals from soils and root exudates

**Figure 4:**
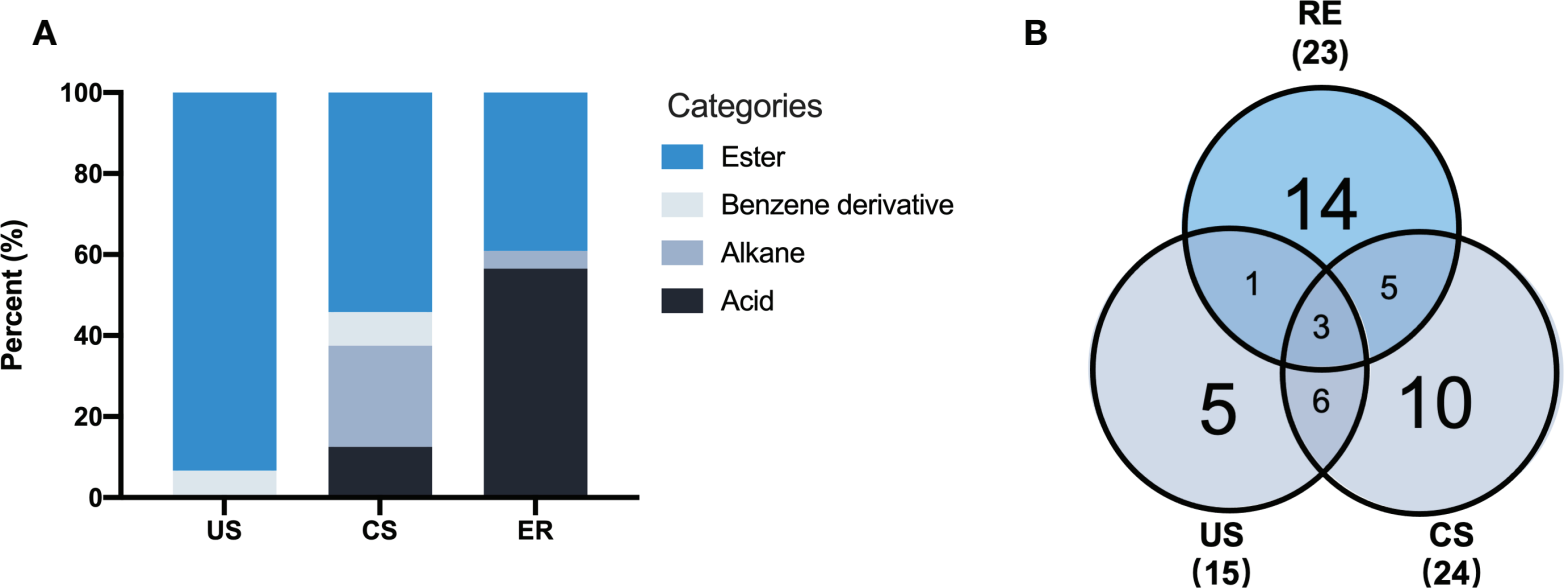
Categories of the metabolites identified from the soils and root exudates by GC-MS analysis **(A)**, The Venn diagram shows the number of metabolites detected in soils and root exudates samples **(B)**. The Numbers in brackets represent the total number of identified metabolites in each group. Additional CS-MS chart can be found in **Supplementary Material**.

Herein, three rhizosphere soil samples (CS) were obtained from the continuously cultivated soil of *P. notoginseng* for three years, and three uncultivated soil samples (US) were obtained from the adjacent field. Moreover, three samples (RE) were extracted from exudates of *P. notoginseng* adsorbed on AC and added to the nutrient solution. A total of 44 different components with more than 80% similarity value were identified from all samples based on the GC-MS analysis (**Table 3**). The identified components were of four types: 14 acids (32%), 22 esters (50%), 6 alkanes (13%), and 2 benzene derivatives (5%). By comparing the types of compounds in the two soil extracts (**Figure 4**), continuous cropping soil contains more acid compounds than uncultivated soil. However, RE contains the most types of acids compounds.

**Table 3:** The list of the identified components in the soils and root exudates of *P. notoginseng*.

Peaks ID	Compounds	CAS	RT (min)	Source ^*^	Classification
1	Benzoic acid	65-85-0	12.863	RE	Acid
2	o-Toluic acid	118-90-1	14.599	RE	Acid
3	Ethyl caproate	123-66-0	14.796	RE	Ester
4	m-Toluic acid	99-04-7	15.093	RE	Acid
5	2,5-Dimethylbenzaldehyde	5779-94-2	15.171	CS	Benzene derivative
6	p-Toluic acid	99-94-5	15.29	RE	Acid
7	Phthalic acid	88-99-3	16.421	RE	Acid
8	2-Hydroxyoctanoic acid	617-73-2	17.115	RE	Acid
9	2-Propionylbenzoic acid	2360-45-4	17.887	CS	Acid
10	3-methylphthalic acid	37102-74-2	18.495	RE	Acid
11	4-Methylphthalic acid	4316-23-8	19.356	RE	Acid
12	Dimethyl phthalate	131-11-3	20.608	US, RE	Ester
13	2,4-Di-tert-butylphenol	96-76-4	21.917	US, CS	Benzene derivative
14	Heptadecane	629-78-7	22.459	CS	Alkane
15	1-Fluorododecane	334-68-9	26.091	CS	Alkane
16	Hexadecane	544-76-3	26.169	CS	Alkane
17	Tetradecanoic acid	544-63-8	26.524	RE	Acid
18	Methyl tetradecanoate	124-10-7	26.767	US, CS	Ester
19	Methyl 3-(3-hydroxyphenyl) acrylate	3943-95-1	27.377	CS	Ester
20	Methyl 4-hydroxycinnamate	3943-97-3	27.391	CS	Ester
21	Methyl tetracosanoate	2442-49-1	28.101	US, CS	Ester
22	Methyl pentadecanoate	7132-64-1	28.127	US	Ester
23	Methyl 9-methyltetradecanoate	213617-69-7	28.899	US	Ester
24	Methyl hexacosanoate	5802-82-4	30.183	US	Ester
25	(Z)-Methyl hexadec-9-enoate	1120-25-8	30.49	US	Ester
26	Dibutyl phthalate	84-74-2	30.54	US, CS, RE	Ester
27	Palmitic acid	57-10-3	30.605	CS, RE	Acid
28	11-Hexadecenoic acid methyl ester	55000-42-5	30.679	US	Ester
29	Methyl palmitate	112-39-0	30.898	US, CS, RE	Ester
30	Butyl PalMitate	111-06-8	31.571	CS, RE	Ester
31	Ethyl palmitate	628-97-7	32.23	CS, RE	Ester
32	Elaidic acid	112-79-8	33.883	RE	Acid
33	Oleic Acid	112-80-1	33.986	RE	Acid
34	methyl linoleate	112-63-0	34.072	US, CS	Ester
35	Methyl oleate	112-62-9	34.191	US, CS	Ester
36	2-Octylcyclopropanedodecanoic acid methyl ester	10152-65-5	34.211	US, CS	Ester
37	Stearic acid	57-11-4	34.337	CS, RE	Acid
38	Methyl stearate	112-61-8	34.684	US, CS, RE	Ester
39	Ethyl stearate	111-61-5	34.902	RE	Ester
40	Bis(2-ethylhexyl) Fumarate	141-02-6	35.455	RE	Ester
41	Heptacosane	593-49-7	39.32	CS	Alkane
42	Diethyl sulfite	623-81-4	40.896	CS	Ester
43	Tetracosane	646-31-1	42.418	CS, RE	Alkane
44	9-Butyldocosane	55282-14-9	43.881	CS	Alkane

* US represents the uncultivated soil;CS represents the continuously cultivated soil;RE represents the root exudates from hydroponics.

**Figure 5:**
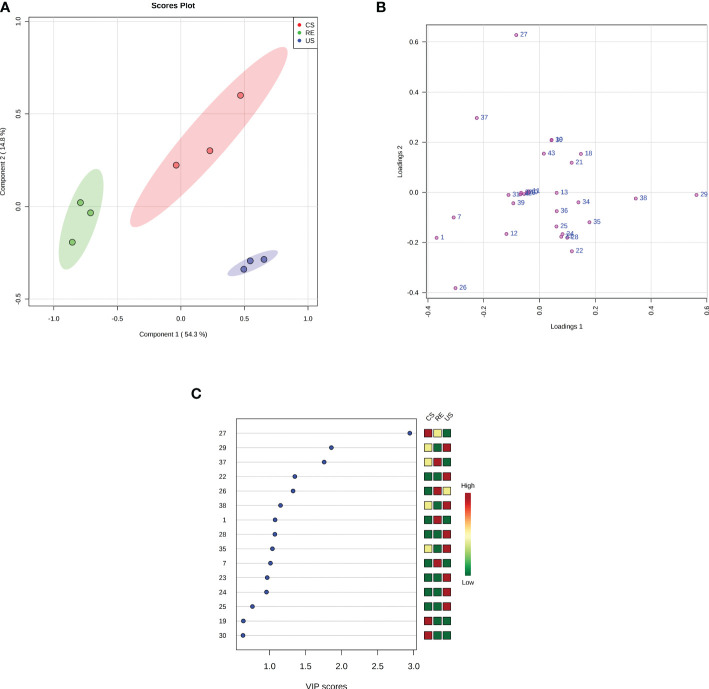
Multivariate analysis using PLS-DA. **(A)** PLS-DA score plots. The explained variances are shown in brackets; **(B)** PLS-DA loadings plot. The numbers in the loading plot of all the samples correspond to the Peak ID provided in **Table 3**; **(C)** Important features identified by PLS-DA. The colored boxes on the right indicate the relative concentrations of the corresponding metabolite in each group under study. The numbers on the left correspond to the Peak ID provided in **Table 3**.

As shown in **Figure 5**, the RE, CS, and US were clearly separated by PC1 and PC2 in the score plot. Based on the loading plot and the VIP value, 12 compounds, were found to play key roles in the classification. In the CS or RE grouping, the relative concentrations of the four compounds palmitic acid (27), stearic acid (37), benzoic acid (1), and phthalic acid (7) are higher than those in the US, and benzoic acid and phthalic acid are unique substances in the RE samples. Therefore, we selected these 4 compounds for activity testing to test their self-toxic activity against *P. notoginseng*.

### 3.5 Bioassay with the identified allelochemicals

Seedling growth bioassays were used to evaluate the allelopathic potential of the identified chemicals at different concentrations. The autotoxicity of the allelochemicals was assayed for growth parameters of *P. notoginseng* at several concentrations (**Table 4**). The benzoic acid, phthalic acid, palmitic acid, and stearic acid significantly inhibited the growth of *P. notoginseng* seedlings at a concentration of 1000 μM. The concentration of the test solution is basically positively correlated with inhibition. Benzoic acid at 1000 μM significantly reduced plant length, root length, and the number of lateral roots to 16, 31, and 42% of those of the control, respectively. When *P.notoginseng* was grown in the nutrient solution containing phthalic acid or palmitic acid, the number of lateral roots was significantly reduced to 24, and 18% compared with those of the control, respectively, even at a low concentration (10 μM). Obviously, these substances more significantly affect the growth of lateral roots. All compounds (at 100 and 1000 μM) decreased the number of lateral roots significantly.

**Table 4:** Effects of the identified chemicals at different concentrations on the growth of *P. notoginseng*.

Allelochemicals	Conc.(μM)	PlantLength(cm)	RootLength(cm)	No. oflateral roots
None (control)	0	6.98 a	4.00 a	6.2 a
Benzoic acid	10	6.43 ab	3.81 ab	6.7 a
	100	6.32 ab	3.32 abcd	5.0 bc
	1000	5.88 b	2.76 cd	3.6 de
Phthalic acid	10	6.43 ab	3.80 ab	4.7 cd
	100	5.86 b	3.46 abcd	3.3 e
	1000	5.87 b	2.62 d	3.1 e
Palmitic acid	10	6.19 ab	3.54 ab	5.1 bc
	100	6.77 ab	3.36 abc	4.5 cd
	1000	6.06 ab	3.06 bcd	3.3 e
Stearic acid	10	6.73 ab	3.90 ab	6.0 ab
	100	6.10 ab	3.02 bcd	3.8 de
	1000	4.90 c	2.88 cd	4.2 ced

Values in a column followed by a different letter differ significantly by Duncan’s test (p < 0.05).

## 4 Discussion

Our results demonstrated that autotoxicity is a factor in the replant failure of *P. notoginseng*. Many factors are considered to be responsible for crop succession disorders (Bouhaouel et al., 2019; Yan et al., 2019). Autotoxicity was reported to be one of the main factors causing continuous cropping obstacles (Singh et al., 1999). Our results show that the water extract of continuous cropping soil has autotoxicity on *P. notoginseng*. Previous studies showed that *P. notoginseng* had a lower germination rate and index when replanted in heavy cropping soil. The EtOAc extract of P. notoginseng soil showed the most significant inhibition of germination rate, germination index, and root elongation of P. notoginseng seeds (PeiJin et al., 2009). The presence of allelochemicals in the soil affects the growth of plants (Guo et al., 2016; Xin et al., 2019). Besides, the extracts of P. notoginseng soil have been found to have chemosensitizing on other plants, such as cabbage, radish, and lettuce (Liu et al., 2019; Ye et al., 2019). In summary, this may be caused by the accumulation of certain allelochemicals in the soil (He et al., 2019; Meng et al., 2022).

In the hydroponic experiment, the addition of AC to the nutrient solution can significantly increase the weight of the roots of *P. notoginseng*, indicating that root exudates have a greater impact on the roots. The content of hydrogen peroxide, peroxidase activity, and malondialdehyde in the leaves of P. notoginseng cultured without the addition of activated carbon were all higher than those of the other, but the difference was not significant. It has been reported that allelochemicals can significantly reduce the enzyme activity of hydrogen peroxide and peroxide in tomato leaves (Staszek et al., 2021). In this study, the allelochemicals had no significant effect on the enzyme activity, which may be due to the insufficient concentration of root exudates in the hydroponic environment. However, it is inferred from the overall trend that with the increase in the concentration of root exudates of P. notoginseng, hydrogen peroxide, and peroxide the activity of bio-enzymes may also increase significantly, which can eliminate excessive hydrogen peroxide to protect the cell (Asgher et al., 2021; Zhang et al., 2021). These data demonstrate that the presence of potential autotoxic factors in the soil will affect the growth of P. notoginseng ginseng.Several types of chemicals have been associated with autotoxicity, including terpenoids, phenolics, steroids, alkaloids, and cyanogenic glycosides (Bruce and Richardson, 1988).In recent years, many of studies have proved that organic acids can significantly inhibit the germination of seeds and the growth of seedlings (Asao et al., 2003). Besides, some ginsenosides have been considered that cause the autotoxicity of *P. notoginseng* (Yang et al., 2015). This study used GC-MS to analyze the key allelochemicals in soil extracts and root exudates. The rhizosphere soil has many organic acids and their derivatives than uncultivated soil. Benzoic acid, phthalic acid, palmitic acid, and stearic acid were found in rhizosphere soil and hydroponic root exudates. These substances are similar to the allelochemicals reported in other plants (Md. Asaduzzaman, 2012; Qiao et al., 2019; Yang et al., 2019). It shows that these substances may also be self-toxic substances with significant self-toxic activity in the root exudates of *P. notoginseng*. In this study, it can be seen from the ion maps of soil extracts and root exudates that the substance dibutyl phthalate was detected in the three samples, and the relative content was more than 30%. Some researcher believe that this substance belongs to root exudates. Another part of the researchers believes these substances are pollutants (Deng et al., 2017; Ge et al., 2020). Whether dibutyl phthalate is notoginseng root exudates remains to be further verified.

Some identified chemicals showed autotoxicity against seed germination and seedling growth at a certain concentration. The benzoic acid, phthalic acid, palmitic acid, and stearic acid significantly affected the root growth of *P. notoginseng*. Previous studies have shown that allelochemicals exert autotoxic effects on *P. notoginseng* seed germination at a concentration of 10-1000μM. Therefore, we chose this concentration for the bioassay. In practice, some allelochemicals are present in lower concentrations and have no effect on plants, but allelopathy occurs (Cheng and Cheng, 2015). This experiment demonstrated the autotoxicity of a single allelochemical, which is in consistent with the actual field conditions. Although this concentration may not be related to natural conditions, our experiment was designed to test the causal relationship. Of course, our work has some limitations, and additional studies are needed to assess the interaction between autotoxicity and other factors of replanting failures, such as soil-borne pathogens, deterioration of soil physicochemical properties, and soil microbial community imbalance. These studies could be used to understand the mechanism of replantation failure of *P. notoginseng*.

## 5 Conclusions

The results showed that the growth of *P. notoginseng* was inhibited by the root exudates, and the AC has the function of adsorbing allelochemicals. The potential allelochemicals were detected as benzoic acid, phthalic acid, salicylic, palmitic acid, and stearic acid. In summary, allelochemicals accumulate in the rhizosphere during the course of root secretion or degradation, etc. and exhibit autotoxicity to *P. notoginseng* when they reach a certain concentration.

## Data availability statement

The original contributions presented in the study are included in the article/**Supplementary Material**. Further inquiries can be directed to the corresponding authors.

## Author contributions

WX designed the research. WX, JC, FZ, RH and LL performed the experiments and analyzed the data. WX wrote the draft of the manuscript. LL and RH reviewed the draft. All authors contributed to the article and approved the submitted version.
