# Supplementary material for: Autotoxicity in Panax notoginseng of root exudatesand their allelochemicals
Source: Front Plant Sci. 2022 Dec 20;13:1020626. doi: 10.3389/fpls.2022.1020626 (PMC9807909; doi:10.3389/fpls.2022.1020626)
Supplement: Supplementary file 1 [file DataSheet_1.pdf]

# Autotoxicity in *Panax notoginseng* of Root Exudates and their Allelochemicals

**Wei Xiang [1,2,3], Jianhua Chen[3], Fengyuan Zhang[3], Rongshao Huang [1*] and Liangbo Li [1*]**

[1]College of Pharmacy, Guangxi University of Chinese Medicine, Nanning, China

[2]College of Horticulture, Hunan Agricultural University, Changsha, China

[3]College of Agriculture, Guangxi University, Nanning, China

*** Correspondence:**
Rongshao Huang,
hrshao802@163.com (R.H.)
Liangbo Li
llb100@126.com (L.L.)

**Supplementary Table 1.** Relative peak area percentage of total (%) for 44 identified compounds[*].

| Peaks ID | US-1 | US-1 | US-1 | CS-1 | CS-2 | CS-3 | RE-1 | RE-2 | RE-3 |
|---|---|---|---|---|---|---|---|---|---|
| 1 | - | - | - | - | - | - | 23.49 | 13.95 | 3.36 |
| 2 | - | - | - | - | - | - | 0.36 | 1.04 | - |
| 3 | - | - | - | - | - | - | - | 1.29 | - |
| 4 | - | - | - | - | - | - | 0.49 | 1.54 | - |
| 5 | - | - | - | - | 10.44 | - | - | - | - |
| 6 | - | - | - | - | - | - | 0.23 | 0.73 | - |
| 7 | - | - | - | - | - | - | 12.23 | 15.98 | 1.31 |
| 8 | - | - | - | - | - | - | - | 1.11 | - |
| 9 | - | - | - | - | 1.22 | - | - | - | - |
| 10 | - | - | - | - | - | - | 0.22 | 0.92 | - |
| 11 | - | - | - | - | - | - | - | 0.90 | - |
| 12 | 0.84 | 0.88 | 0.88 | - | - | - | 5.47 | - | 2.33 |
| 13 | 2.37 | 1.93 | 1.84 | - | 7.93 | - | - | - | - |
| 14 | - | - | - | - | 1.20 | - | - | - | - |
| 15 | - | - | - | - | 1.44 | - | - | - | - |
| 16 | - | - | - | - | 1.11 | - | - | - | - |
| 17 | - | - | - | - | - | - | - | 1.07 | - |
| 18 | 2.01 | 1.79 | 1.94 | 3.82 | - | 3.22 | - | - | - |
| 19 | - | - | - | 2.84 | - | - | - | - | - |
| 20 | - | - | - | - | 3.44 | - | - | - | - |
| 21 | 1.27 | 1.11 | 1.22 | 2.30 | - | 2.07 | - | - | - |
| 22 | 2.44 | 2.20 | 2.39 | - | - | - | - | - | - |
| 23 | 0.91 | 2.89 | 0.89 | - | - | - | - | - | - |

| | | | | | | | | | |
|---|---|---|---|---|---|---|---|---|---|
| 24 | 1.36 | 1.22 | 1.12 | - | - | - | - | - | - |
| 25 | 0.21 | 1.33 | 1.46 | - | - | - | - | - | - |
| 26 | 37.34 | 44.39 | 42.72 | 24.53 | 43.26 | 43.07 | 43.33 | 44.06 | 69.70 |
| 27 | - | - | - | 11.34 | 7.56 | 11.45 | 1.79 | 7.50 | 8.54 |
| 28 | 3.97 | 0.97 | 1.07 | - | - | - | - | - | - |
| 29 | 29.13 | 25.62 | 28.51 | 27.33 | 9.20 | 23.06 | 1.49 | - | 1.54 |
| 30 | - | - | - | 2.80 | - | - | - | - | - |
| 31 | - | - | - | - | 1.87 | - | 2.18 | - | 2.50 |
| 32 | - | - | - | - | - | - | - | 0.96 | 0.89 |
| 33 | - | - | - | - | - | - | - | 1.38 | - |
| 34 | 2.18 | 2.17 | 1.45 | 1.66 | - | 1.56 | - | - | - |
| 35 | 3.82 | 2.94 | 3.20 | 2.09 | - | 1.86 | - | - | - |
| 36 | 1.09 | 0.90 | 0.99 | - | 1.62 | - | - | - | - |
| 37 | - | - | - | 5.60 | 3.85 | 5.20 | 6.68 | 6.23 | 7.66 |
| 38 | 11.07 | 9.66 | 10.32 | 8.61 | 5.88 | 8.52 | - | - | - |
| 39 | - | - | - | - | - | - | 1.36 | - | 2.16 |
| 40 | - | - | - | - | - | - | - | 1.33 | - |
| 41 | - | - | - | 1.56 | - | - | - | - | - |
| 42 | - | - | - | 2.09 | - | - | - | - | - |
| 43 | - | - | - | 1.96 | - | - | 0.68 | - | - |
| 44 | - | - | - | 1.48 | - | - | - | - | - |

* The data of each compound were the average of three parallel samples and normalized by peak area normalization method. US represents the uncultivated soil; CS represents the continuously cultivated soil; RE represents the root exudates from hydroponics. -: without detection.

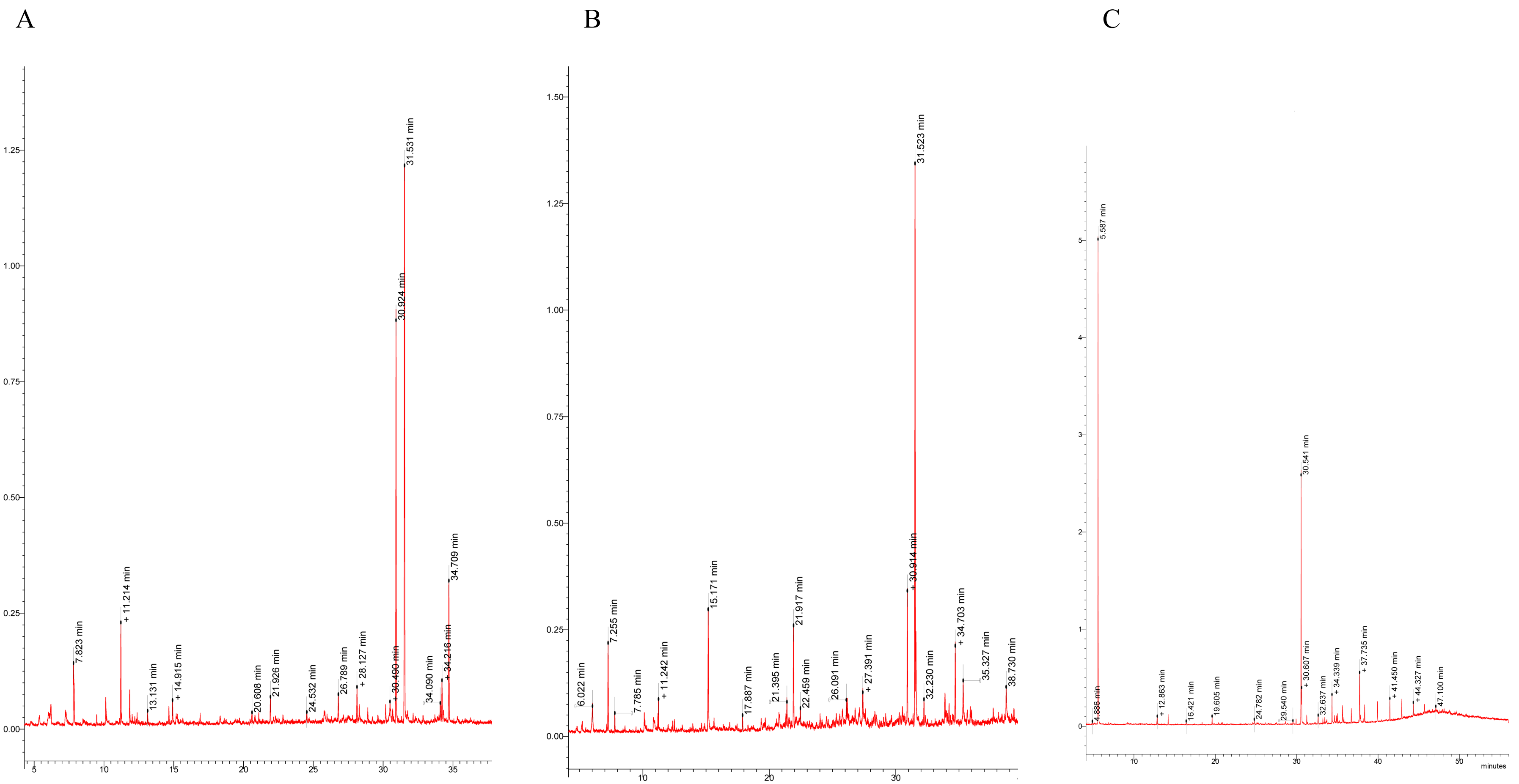


**Supplementary Figure 1.** GC-MS total ion chromatograms (TIC) of the soil and root exudates sample.

(A) uncultivated soil (B) continuously cultivated soil (C) root exudates from hydroponics.
